# Genotypic testing on HIV-1 DNA as a tool to assess HIV-1 co-receptor usage in clinical practice: results from the DIVA study group

**DOI:** 10.1007/s15010-013-0510-3

**Published:** 2013-10-22

**Authors:** V. Svicher, C. Alteri, M. Montano, A. Nori, R. D’Arrigo, M. Andreoni, G. Angarano, A. Antinori, G. Antonelli, T. Allice, P. Bagnarelli, F. Baldanti, A. Bertoli, M. Borderi, E. Boeri, I. Bon, B. Bruzzone, R. Barresi, S. Calderisi, A. P. Callegaro, M. R. Capobianchi, F. Gargiulo, F. Castelli, R. Cauda, F. Ceccherini-Silberstein, M. Clementi, A. Chirianni, M. Colafigli, A. D’Arminio Monforte, A. De Luca, A. Di Biagio, G. Di Nicuolo, G. Di Perri, F. Di Santo, G. Fadda, M. Galli, W. Gennari, V. Ghisetti, A. Costantini, A. Gori, R. Gulminetti, F. Leoncini, G. Maffongelli, F. Maggiolo, R. Maserati, F. Mazzotta, G. Meini, V. Micheli, L. Monno, C. Mussini, S. Nozza, S. Paolucci, G. Palù, S. Parisi, G. Parruti, A. R. Pignataro, T. Quirino, M. C. Re, G. Rizzardini, M. Sanguinetti, R. Santangelo, R. Scaggiante, G. Sterrantino, O. Turriziani, M. L. Vatteroni, C. Viscoli, V. Vullo, M. Zazzi, A. Lazzarin, C. F. Perno

**Affiliations:** 1 Department of Experimental Medicine, University of Rome “Tor Vergata”, Via Montpellier 1, 00133 Rome, Italy; 2 Sequencing and Antiviral Drug Monitoring Unit, INMI “L. Spallanzani”, Rome, Italy; 3 Clinic of Infectious Diseases, Bari University, Bari, Italy; 4 “Sapienza” University of Rome, Rome, Italy; 5 Microbiology and Virology Laboratory, University of Turin, Turin, Italy; 6 Department of Biochemical Sciences and Public Health, Marche Politechnic University Medical School, Ancona, Italy; 7 Department of Virology and Microbiology, Molecular Virology Unit, Fondazione IRCCS Policlinico San Matteo, Pavia, Italy; 8 Section of Microbiology of the Department of Hematology and Oncologic Science, University of Bologna, Bologna, Italy; 9 Laboratory of Microbiology and Virology, Vita-Salute San Raffaele University, Milan, Italy; 10 San Martino Hospital, Genoa, Italy; 11 Department of Infectious Diseases, Ospedali Riuniti, Bergamo, Italy; 12 Ospedali Civili di Brescia, Brescia, Italy; 13 Institute of Clinical Infectious Diseases, Catholic University of Sacred Heart, Rome, Italy; 14 III Division of Infectious Diseases, A.O.R.N. Cotugno, Naples, Italy; 15 “S. Paolo” Hospital, Milan, Italy; 16 UOC Malattie Infettive Universitarie, Azienda Ospedaliera Universitaria Senese, Siena, Italy; 17 “S.M. Annunziata” Hospital, Florence, Italy; 18 Institute of Microbiology, Catholic University of Sacred Heart, Rome, Italy; 19 Institute of Infectious and Tropical Diseases, “L. Sacco Hospital”, Milan, Italy; 20 Unit of Microbiology, Modena University Hospital, Modena, Italy; 21 Division of Infectious Diseases, Department of Internal Medicine, San Gerardo Hospital, University of Milano-Bicocca, Monza, Italy; 22 “Careggi” Hospital, Florence, Italy; 23 Department of Biotechnology, University of Siena, Siena, Italy; 24 Clinic of Infectious Diseases, University of Bari, Bari, Italy; 25 Department of Infectious Diseases, “S. Raffaele” Scientific Institute, Milan, Italy; 26 Department of Histology Microbiology and Medical Biotechnology, Padova University, Padua, Italy; 27 Infectious Disease Unit, Pescara General Hospital, Pescara, Italy; 28 Busto Arsizio Hospital, Busto Arsizio, Italy; 29 Virology Unit AOU Pisana, Pisa University, Pisa, Italy; 30 Università degli Studi di Brescia, Brescia, Italy

**Keywords:** HIV DNA, Tropism, Virologically suppressed patients, CCR5 antagonists, Geno2pheno

## Abstract

**Purpose:**

We have developed a sequencing assay for determining the usage of the genotypic HIV-1 co-receptor using peripheral blood mononuclear cell (PBMC) DNA in virologically suppressed HIV-1 infected patients. Our specific aims were to (1) evaluate the efficiency of V3 sequences in B versus non-B subtypes, (2) compare the efficiency of V3 sequences and tropism prediction using whole blood and PBMCs for DNA extraction, (3) compare the efficiency of V3 sequences and tropism prediction using a single versus a triplicate round of amplification.

**Results:**

The overall rate of successful V3 sequences ranged from 100 % in samples with >3,000 copies HIV-1 DNA/10^6^ PBMCs to 60 % in samples with <100 copies total HIV-1 DNA /10^6^ PBMCs. Analysis of 143 paired PBMCs and whole-blood samples showed successful V3 sequences rates of 77.6 % for PBMCs and 83.9 % for whole blood. These rates are in agreement with the tropism prediction obtained using the geno2pheno co-receptor algorithm, namely, 92.1 % with a false-positive rate (FPR) of 10 or 20 % and of 96.5 % with an FPR of 5.75 %. The agreement between tropism prediction values using single versus triplicate amplification was 98.2 % (56/57) of patients using an FPR of 20 % and 92.9 % (53/57) using an FPR of 10 or 5.75 %. For 63.0 % (36/57) of patients, the FPR obtained via the single amplification procedure was superimposable to all three FPRs obtained by triplicate amplification.

**Conclusions:**

Our results show the feasibility and consistency of genotypic testing on HIV-1 DNA tropism, supporting its possible use for selecting patients with suppressed plasma HIV-1 RNA as candidates for CCR5-antagonist treatment. The high agreement between tropism prediction by single and triple amplification does not support the use of triplicate amplification in clinical practice.

## Introduction

Human immunodeficiency virus-1 (HIV-1) enters the target cell using CD4 molecules as primary receptors and either one of two chemokine receptors, CCR5 or CXCR4. On the basis of co-receptor usage, HIV-1 strains are classified as CCR5-, CXCR4- or dual/mixed tropic. The introduction into clinical practice of the CCR5 antagonist maraviroc has required the testing of HIV tropism in each patient prior to therapy [1].

Among the different approaches for tropism determination, genotypic population sequencing predicts HIV co-receptor usage based on the sequence of the V3 loop of HIV-1 gp120. This test has been shown to be a valuable tool in clinical routine and has been incorporated into several guidelines to predict viral tropism [1, 2]. To date, the performances of various genotypic tropism tests have been evaluated in RNA samples from viremic patients [3–7]. Less information is available on the efficiency of V3 sequencing of HIV-DNA from patients with suppressed viremia. Assessing this efficiency has important clinical implications since it would allow maraviroc to be considered part of the switch or simplification strategies in drug-treated patients with undetectable plasma HIV-1 RNA. In addition, as shown in maraviroc clinical trials, a potential immune benefit of this drug might encourage its use as part of intensification strategies in HIV-1-infected patients with impaired CD4 gains despite prolonged suppression of HIV replication with antiretroviral therapy (ART) [8].

Given this background and within the framework of the DIVA (DNA Tropism Italian Validation Concerted Action) group (Appendix 1), we have developed a genotypic tropism test on HIV-DNA for routine clinical diagnostic laboratory testing. In an earlier study, this test was subjected to an inter-laboratory validation procedure [9]. Here, our specific aims are to (1) evaluate the efficiency of V3 sequencing in B versus non-B subtypes, (2) compare the efficiency of V3 sequencing and tropism prediction using whole blood and peripheral blood mononuclear cells (PBMCs) for DNA extraction, and (3) compare the efficiency of V3 sequencing and tropism prediction using a single versus a triplicate round of amplification.

## Materials and methods

### Patients

Contemporary plasma and whole-blood samples were collected from 253 HIV-1 infected highly active ART (HAART)-treated patients with plasma HIV-1 RNA of <50 copies/ml who were followed in 18 centers participating in the DIVA study group. All patients were naïve to maraviroc.

### Viral amplification and sequencing

HIV-1 DNA was extracted from whole blood or PBMCs using the QIAamp DNA Blood Mini kit and QIAamp DNA Mini kit, respectively, according to the manufacturer’s instructions (Qiagen, Hilden, Germany). PBMCs were obtained by separation through a Ficoll-Hypaque gradient as previously described [10].

Amplification of the V3-containing region of the* env *gene consisted of two amplification steps using primers designed on the basis of the gp120 consensus B sequence (downloaded from Los Alamos HIV Database: http://www.hiv.lanl.gov/cgi-bin/NEWALIGN/align.cgi). The forward primer ENVS10 [5′-CCAATTCCCATACATTATTGT-3′; nucleotide (nt) 538–558 of the HIV-1 gp120* env* gene] and the reverse primer V3AS5 (5′-CTTCTCCAATTGTCCCTCA-3′; nt 1,292–1,310) were used for the first amplification step, while the inner forward primer V3S2 (5′-CAGCACAGTACAATGTACACA-3′; nt 630–650) and V3AS5 were used for the second one. The length of the amplicon produced, including the V3 sequence, is 660 nt.

The conditions for the first amplification were one cycle at 93 °C for 12 min, 40 cycles at 93 °C for 30 s, 50 °C for 30 s, and 72 °C for 50 s, with a final step at 72 °C for 10 min. The total reaction volume (40 μl) contained the following master mix: 5 μl of Taq buffer 10×, 3 μl of 25 mM MgCl_2_, 28.95 μl of DNase- and RNase-free bidistilled water, 0.75 μl of 10 μM primers, 0.8 μl of 12.5 mM dNTPs, 0.75 μl of Taq (5 U/μl). The amplification conditions for the semi-nested PCR were one cycle at 93 °C for 12 min, 40 cycles at 93 °C for 30 s, 51 °C for 30 s, and 72 °C for 50 s, with a final step at 72 °C for 10 min. The total reaction volume (45 μl) contained the following master mix: 5 μl of Taq Gold PE buffer 10×, 3 μl of 25 mM MgCl_2_, 33.95 μl of DNase- and RNase-free bi-distilled water, 0.75 μl of 10 μM primers, 0.8 μl of 12.5 mM dNTPs, 0.75 μl of Taq (5 U/μl).

The PCR product was purified using the Microcon PCR purification kit (Millipore Corp., Billerica, MA). Negative and positive control samples were included in each PCR run to exclude false-positive and false-negative reactions. PCR products were then sequenced using the BigDye Terminator v.3.1 Cycle Sequencing kit (Applied Biosystems, Foster City, CA) and an automated sequencer (ABI-3,100; Applied Biosystems). Four different overlapping sequence-specific primers were used to ensure coverage of the V3-sequence by at least two sequence segments. The sequencing conditions were one cycle at 96 °C for 3 min, 25 cycles at 96 °C for 30 s, 50 °C for 10 s, and 60 °C for 4 min), and the following primers were used: V3S6 (5′-CTGTTAAATGGCAGTCTAGC-3′), V3S5 (5′-GTTAAATGGCAGTCTAGCAG-3′), V3AS1 (5′-GAAAAATTCCCCTCCACAATT-3′), and V3AS3bis (5′-CAATTTCTGGGTCCCCTC-3′).

The Siemens sequencing kit was used in three of the centers participating in the DIVA project. In particular, CLIP sequencing was performed using the Trugene Core kit according to the manufacturer’s instructions. The four CLIP reaction mixture contained 2.8 μl of CLIP buffer, 8.8 μl of molecular water, 2.8 μl of forward primer V3S6 (5′-Cy5.5-CTGTTAAATGGCAGTCTAGC-3′) and reverse primer V3AS3bis (5′-Cy5-5′CAATTTCTGGGTCCCCTC GGT-3′) (3 μM), 5 μl of sample cDNA, 3 μl of the four terminator nucleotides, and 4.4 μl of Thermo Sequenase (GE Healthcare Life Sciences, UK) enzyme diluted 1:10 (32 U/μl). The CLIP cycling profile was 5 min at 94 °C, followed by 30 cycles of 20 s at 94 °C, 20 s at 55.5 °C, and 60 s at 70 °C, with a final extension of 7 min at 70 °C and 30 min at 4 °C. Thereafter, Stop Loading Dye (6 μl) was added. Samples were heated to 94 °C for 3 min and incubated at 4 °C. Fragments were separated on a TruGene Tower (Siemens) with a 6 % polyacrylamide gel. Sequence data were acquired and analyzed using the OpenGene DNA Sequencing System (Siemens) and read against a V3 loop sequence-specific reference.

For each sample, HIV-1 subtype was determined by phylogenetic analysis of the related pol nucleotide sequences. Phylogenetic analysis of V3 sequences was also used to identify potential cross-contaminations during the process.

To compare tropism prediction via single or triplicate amplification, DNA extracted from whole blood was divided into four aliquots. One aliquot was randomly chosen to be processed by a single amplification. Each of the remaining three aliquots underwent a process of V3 amplification and sequencing.

### Genotypic prediction of viral tropism

HIV-1 co-receptor usage was inferred from the V3 nucleotide sequence by using the geno2pheno algorithm (http://coreceptor.bioinf.mpi-inf.mpg.de/) and by using the clonal version of geno2pheno set at a false-positive rate (FPR) of 20 %, as proposed by the current guidelines for tropism determination [1], and 5.75 and 10 %.

For the triple amplification, an isolate was predicted as CXCR4-tropic if at least one of the three tests reported it to be CXCR4-tropic.

### Quantification of total HIV-1 DNA

To quantify total HIV-1 DNA in PBMCs, we adapted the Real Time TaqMan protocol published by Viard et al. [11] the LightCycler system (Roche Molecular Biochemicals, Indianapolis, IN). The methodology is describd in detail in Appendix 2.

## Results

### Patient’s characteristics

This study included a total of 253 HIV-1 infected HAART-treated patients with undetectable plasma HIV-1 RNA (<50 copies/ml). The clinical and viro-immunological characteristics of patients are shown in Table 1. Median time of virological success was 3.5 [interquartile range (IQR) 2.1–5.9] years. At the time of sample collection, the median CD4 cell count was 578 (IQR 416–780) cells/μl.

**Table 1:** Patients’ characteristics

Characteristics	Overall samples^a^ (*N* = 253)	Amplified samples^a^ (*N* = 201)	Not amplified samples^a^ (*N* = 52)	*P* value
Male, *N* (%)	148 (72.2)	123 (74.1)	25 (64.1)	ns
Italians, *N* (%)	158 (91.9)	129 (93.5)	29 (85.3)	ns
Risk factors, *N* (%)				
Heterosexual	60 (42.9)	48 (42.1)	12 (46.1)	ns
Homosexual	38 (27.1)	30 (26.3)	6 (23.1)	ns
Drug addiction	42 (30)	36 (31.6)	8 (30.8)	ns
Age, median (IQR)	49 (42–55)	48 (43–54)	46 (43–58)	ns
Drug-experienced patients, *N* (%)	156 (94.0)	120 (97.6)	36 (83.7)	0.003
HIV-1 subtypes, *N* (%)^b^				
B	100 (75.2)	78 (73.6)	22 (81.5)	ns
CRF02_AG	5 (3.8)	5 (4.7)	0 (0.0)	ns
C	6 (4.5)	6 (5.7)	0 (0.0)	ns
F	9 (6.8)	7 (6.6)	2 (7.4)	ns
Others	13 (9.8)	10 (9.4)	3 (11.1)	ns
Year of starting current therapy, median (IQR)	2009 (2007–2010)	2009 (2007–2010)	2009 (2008–2010)	ns
Virological suppression time (year), median (IQR)	3.5 (2.1–5.9)	3.2 (1.9–5.4)	2.9 (2.1–5.8)	ns
Current therapy, *N* (%)				
Non-nucleoside RT inhibitors	67 (34.3)	46 (31.5)	21 (42.8)	ns
Protease inhibitors	86 (44.1)	70 (47.9)	16 (32.6)	ns
Raltegravir	26 (13.3)	23 (15.7)	9 (18.4)	ns
Unknown	16 (8.2)	7 (4.8)	3 (6.1)	ns
Total HIV DNA copies/10e-6 PBMCs, median (IQR)	1,582 (279–5,574)	1,197 (325–2,354)	365 (13–1,812)	ns
HIV RNA, log10, median (IQR)				
Pre-HAART	4.9 (4.0–5.4)	5.0 (4.0–5.4)	4.9 (4.0–5.3)	ns
At V3 sampling date	<1.7	<1.7	<1.7	ns
CD4 cells/mm^3^, median (IQR)				
Pre-HAART	250 (147–380)	261 (138–367)	300 (174–415)	ns
Nadir	211 (110–300)	201 (115–301)	230 (79–335)	ns
At V3 sampling date	578 (416–780)	368 (265–546)	558 (317–670)	0.03

Fisher exact test and Wilcoxon test were used for dichotomic and continuous variables, respectively

*IQR* Interquartile range,* HAART* highly active antiretroviral therapy,* PBMCs* peripheral blood mononuclear cells, *ns* not significant

^a^The table reports both the characteristics of the overall group of patients analyzed (*N* = 253) and stratified in patients with or without successful V3 sequencing

^b^Human immunodeficiency virus-1 (HIV-1) subtypes and recombinant forms were available for 133 patients and were determined by phylogenetic analysis using HIV-a pol sequences

### HIV-1 subtyping

HIV-1 subtyping (available for 133 patients and assessed by phylogenetic analysis based on HIV-1 pol sequences) showed that the large majority of patients harbored HIV-1 B subtype (*N* = 100, 75.2 %). Other non-B subtypes were CRF02_AG (*N* = 5, 3.8 %), C (*N* = 6, 4.5 %), and F (*N* = 9, 6.8 %). For 122 patients, both the HIV-1 pol and V3 sequences were available. HIV-1 subtyping by phylogenetic analysis using either the pol or env sequences was concordant for 86.1 % (105/122) of patients. In 15 patients, pol sequences based phylogenetic analysis predicted the presence of recombinant forms (7/15) or subtype F1(5/15), while V3 sequence-based phylogenetic analysis predicted the presence of B subtypes. In the remaining three cases, phylogenetic analysis based on pol and env sequences predicted the presence of different non-B subtypes. Thus, misclassifications were mainly due to the presence of non-B subtypes or recombinant forms.

### Efficiency of V3 sequencing and tropism prediction

**Fig. 1:**
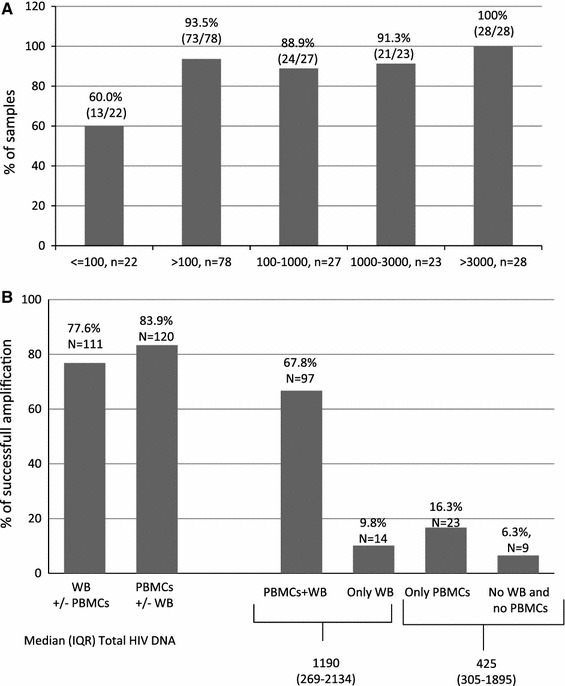
Rate of successful V3 sequencing. ** a** Rate of successful V3 sequencing based on different levels of total HIV-1 DNA quantification. ** b** Rate of successful V3 sequencing in patients for which DNA was extracted from  whole blood (*WB*) or peripheral blood mononuclear cells (*PBMCs*). The quantification of total HIV-DNA is expressed in copies/10^6^ cells

A total of 201 V3 sequences were obtained among the 253 samples analyzed. In order to define the efficiency of V3 sequencing according to the level of total HIV-1 DNA, we analyzed 178 samples for the level of total HIV-1 DNA available. The success rate of V3 sequencing was 93.5 % in samples with >100 copies total HIV-1 DNA/10^6^ PBMCs, reaching 100 % in samples with >3,000 copies total HIV-1 DNA/10^6^ PBMCs. In samples with <100 copies total HIV-1 DNA/10^6^ PBMCs, the success rate of V3 sequencing was 60 % (Fig. 1a). Thus, this methodology for V3 sequencing on proviral DNA performed well, also at a low level of HIV-DNA. No differences in the success rate of V3 sequencing were observed at the three centers using the Siemens methodology.

Among the 33 patients infected with non-B subtypes, V3 sequences were obtained for 28 (84.8 %) patients. V3 sequences were also obtained for atypical recombinants forms such as CFR31 and CRF06.

### Tropism prediction

Among the 201 V3 sequences obtained, CXCR4 co-receptor usage was reported in 35 (17.4 %), 48 (23.9 %), and 68 (33.6 %) of patients using an FPR of 5.75, 10, and 20 %, respectively. A FPR of <5.75 % was associated with a lower CD4 cell count at the time of V3 genotypic tropism testing and with a lower increase in CD4 cell count from the beginning of HAART therapy to the time of V3 sequencing [median (IQR) CD4 cell count: 459 (317–590) for FPR of <5.75 % vs. 519 (360–862) for FPR of 5.75–20 % and 579 (435–755) for FPR of >20 %; median (IQR) gain of CD4 cell count: 20 (−58 to 163) for FPR of <5.75 % vs. 187 (60–281) for FPR of 5.75–20 % and 164 (22–379) for FPR of >20 %]. Interestingly, a lower FPR were also associated with a higher CD8 cell count at the time of V3 genotyping [median (IQR) CD8 cell count: 1,120 (711–1,464) for FPR of <5.75 % vss 760 (641–1,142) for FPR of 5.75−20 % and 745 (587–987) for of FPR >20 %] (Table 2).

**Table 2:** Characteristics of R5 and X4 proviruses in patients from the DIVA project according to the false-positive rate

Characteristics	Patients with FPR			*P* value for trend^a^	*P* value <5.75 vs >5.75^a^
	<5.75 %	5.75–20 %	>20 %		
Viremia at baseline log copies/ml	1.7 (0.7–4.8)	1.7 (1.6–4.5)	3.1 (1.6–4.4)	ns	ns
Pre-HAART Viremia	5.1 (4.0–5.3)	5.1 (4.3–5.3)	5.2 (4.2–5.4)	ns	ns
CD4 cells/μl at time of V3 sequencing	459 (317–590)	519 (360–862)	579 (435–755)	0.06	0.02
CD4 cells/μl at baseline (BL)	336 (249–630)	368 (228–592)	371 (239–498)	ns	ns
CD4 nadir cells/μl	153 (51–292)	216 (151–309)	203 (125–302)	ns	ns
Pre-HAART CD4 cell count cells/μl	257 (97–445)	219 (107–419)	263 (152–350)	ns	ns
Change in CD4 cells/μl					
Between BL and V3 sequencing	20 (−58 to 163)	187 (60–281)	164 (22–379)	0.005	0.001
Between pre-HAART and V3 sequencing	212 (−37 to 301)	314 (206–622)	309 (182–552)	0.01	0.004
CD8 count at V3 sequencing	1,120 (711–1,464)	760 (641–1,142)	745 (587–987)	0.01	0.004
CD8 percentage at V3 sequencing	54 (47–63)	45 (35–55)	40 (34–46)	0.001	<0.001
Time under virological suppression (years)	3.0 (1.7–5.8)	3.9 (2.2–6.4)	3.6 (2.1–5.9)	ns	ns

Data are presented as the median with the interquartile range (IQR) given in parenthesis

*FPR* False-positive rate

^a^Statistically significant difference was calculated by the chi-square test for trend and by the Fisher exact test between patients with an FPR of <5.75 % and those with an FPR of >5.75%

### Comparison of the efficiency of V3 sequencing and tropism prediction using whole blood versus PBMCs for DNA extraction

A total of 143 pairs of PBMCs and whole blood samples obtained from identical collection tubes were processed. The overall rate of successful V3 sequencing was 77.6 % (111/143) and 83.9 % (120/143) for PBMCs and whole blood aliquots, respectively (Fig. 1b). In particular, V3 sequences were obtained from both whole blood and PBMCs in 67.8 % (97/143) of the samples. V3 sequences were obtained from only PBMCs and only whole blood in 16.3 % (*N* = 23) and 9.8 % (*N* = 14) of samples, respectively. For the remaining 6.3 % (*N* = 9) of samples, V3 sequences were not obtained from either PBMCs or whole blood. Samples not amplified and samples successfully amplified only from PBMCs were characterized by a lower total HIV-DNA quantification.

The agreement with the prediction of viral tropism was 92.1 % when an FPR of 10 and 20 % was used. This correspondence increased to 96.5 % with an FPR of 5.75 %. The median similarity between V3 sequences obtained via whole blood and via PBMCs was 99.5 % (IQR 90.6–100). The distribution of FPRs obtained via whole blood and via PBMCs was superimposable, and for 47.4 % (46/97) of patients, the V3 sequences obtained via whole blood and PBMCs had the same FPR value.

### Triplicate versus single amplification procedure

**Fig. 2:**
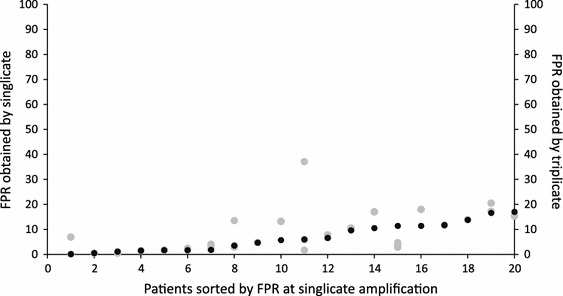
Distribution per patient of the false-positive rate (*FPR*) obtained by single amplification (*black points*) and the three FPRs obtained by triplicate amplification (*grey points*). Only patients with an X4 provirus were included in the graph

Of the 243 samples, 57 were processed by both single and triplicate amplification of the V3 region. The concordance between tropism prediction using the single versus the triplicate amplification was 98.2 % (56/57) of samples/patients using a FPR of 20 % and 92.9 % (53/57) using a FPR of 5.75 or 10 %. For 63.1 % (36/57) of patients/samples, the FPR value obtained via the single amplification procedure was superimposable onto the FPRs obtained by triplicate amplification (Fig. 2), thus indicating a substantial similarity among viral quasispecies. Among the four discordant cases, three resulted in reclassification of CCR5 in CXCR4 tropism. In these three samples, the tropism prediction of the V3 sequences obtained by triplicate amplification always revealed the co-existence of CCR5-using and CXCR4-using strains (Table 3). No relationship was found between the discordant tropism prediction and HIV-1 DNA content (Table 3).

**Table 3:** Overview of reclassified samples using a triplicate tropism procedure

FPR	ID	FPR by single amplification	FPR by triplicate amplification			Tropism reclassification		HIV-DNA copies/10^e−6^ PBMCs
			FPR A	FPR B	FPR C	Singlicate	Triplicate	
5.75 %	13	11.4	4.7	2.8	3.7	R5	X4	1,303
	80	34.3	1.5	1.5	58.7	R5	X4	425
	96	6	5.8	1.7	37.1	R5	X4	Not available
	95	5.7	13.2	13.2	13.2	X4	R5	372
10 %	13	11.4	4.7	2.8	3.7	R5	X4	1,303
	80	34.3	1.5	1.5	58.7	R5	X4	425
	20	19.4	17	7.8	36.4	R5	X4	1,768
	95	5.7	13.2	13.2	13.2	X4	R5	372
20 %	80	34.3	1.5	1.5	58.7	R5	X4	425
Guidelines approach^a^	80	34.3	1.5	1.5	58.7	R5	X4	425
	82	16.6	20.5	20.5	17.2	X4	R5	–
	95	5.7	13.2	13.2	13.2	X4	R5	372
	156	17.3	17.3	18.2	18.2	X4	R5	–
	161	10.5	17	17	17	X4	R5	–
	169	17	15.4	15.4	15.4	X4	R5	–
	177	13.8	13.8	13.8	13.8	X4	R5	–
	182	11.4	18	18	18	X4	R5	–
	223	12	12	12	12	X4	R5	–

The table reports samples with a different tropism prediction using the single or the triplicate amplification procedure. The FPRs used as cut-off for tropism prediction were: 5.75, 10, and 20 %, respectively. For the triple amplification, an isolate was predicted as CXCR4-tropic if at least one of the three tests was reported to be CXCR4-tropic

^a^The European guidelines [1] advise a FPR of 10 % when triplicate amplification is used, and a FPR of 20 % for single amplification

## Discussion

We have described the performance of a population-based V3 sequencing method for tropism prediction on HIV-1 DNA. This assay was effective for HIV-1 tropism determination in patients with undetectable plasma HIV-1 RNA. Indeed, the rate of successful V3 sequencing (using whole blood for DNA extraction) was 93.5 % in samples with >100 copies HIV-1 DNA /10^6^ PBMCs, reaching 100 % in samples with >300 copies HIV-1 DNA/10^6^ PBMCs, a percentage comparable to that observed for V3 sequencing from HIV-RNA samples in viremic patients [6].

The method did not suffer from subtype-related variability within HIV group M subtypes. Indeed, 84.8 % of samples from non-B subtypes were successfully amplified and sequenced. In this study, HIV-1 subtyping was inferred by phylogenetic analysis based on pol sequences. However, we did note some misclassifications when phylogenetic analysis based on env sequences and the geno2pheno algorithm were used to infer HIV-1 subtypes.

In addition, in 15 of the 133 patients with CCR5 tropism, maraviroc had been introduced into their antiretroviral regimen. All of these 15 patients had detectable plasma HIV-1 RNA at week 12, and 12 of the 15 patients had detectable HIV-1 RNA at week 24. All 15 patients maintained a plasma HIV-1 RNA level of <50 copies/ml at both week 12 and 24. Although further clinical evaluation of genotypic tropism testing is needed, this result supports the use of genotypic tropism testing in proviral DNA to select patients as candidates for maraviroc treatment. This is in line with a recent study showing that among 71 patients treated with maraviroc, a plasma HIV-1 RNA of <50 copies/ml was maintained in 85.4 % of cases who had a viral load quantification at month 9 [12].

In this study, CXCR4-using viruses were detected in 17.4, 23.9, and 33.6 % of patients using an FPR of 5.75, 10, and 20 %, respectively. This prevalence is in line with the results reported from two other independent studies in a group of virologically suppressed patients [4, 13]. In these studies, a subset of 78 and 140 HAART-treated patients with suppressed viremia were studied, with CXCR4-using viruses detected in proviral DNA in 34.0 [4] and 30.0 % [13] of patients analyzed, respectively.

This is the first study to evaluate the rate of successful V3 sequencing and tropism prediction using PBMCs and whole blood for DNA extraction. We found a comparable rate of successful V3 sequencing and a high concordance of tropism prediction with the most commonly used FPRs. These results suggest that whole blood can be used in clinical practice for determining HIV-1 DNA co-receptor tropism, thereby eliminating the need and inconvenience of PBMC preparation.

We also addressed the issue of single versus triplicate amplification for tropism determination. A previous study, conducted in 43 HIV-DNA samples from viremic patients, showed that triplicate testing on HIV-1 DNA resulted in the reclassification from CCR5 to CXCR4 tropism in one, two, and four patients using a FPR of 5.75, 10, and 20 %, respectively [14]. A similar reclassification rate was observed in the Italian Cohort of Antiretroviral Naive Patients cohort when triplicate testing was performed using an FPR of 10 % on 42 paired HIV-DNA and -RNA samples from viremic patients [15]. In our group of 57 HIV-DNA samples obtained from virologically suppressed patients, we found a high agreement in tropism prediction using the single versus the triplicate amplification (98.2 % using a FPR of 20 % and 92.9 % using a FPR of 5.75 and 10 %), and for a large proportion of samples the FPR value obtained via the single amplification procedure was superimposable onto those obtained by triplicate amplification. Triplicate amplification resulted in reclassification from CCR5 to CXCR4 tropism for only three, three, and one samples using a FPR of 5.75, 10 and 20 %, respectively. Thus, the high concordance of tropism prediction among samples processed via single and triple amplification suggests that single amplification can be used in diagnostic practice, as also suggested by the 2012 update of the Austrian–German Treatment Guidelines (http://coreceptor.bioinf.mpi-inf.mpg.de/index.php). Indeed, the higher cost and turnaround time associated with triplicate testing do not appear to be justified, at least in virologically suppressed patients.

Recent guidelines suggest using an FPR of 10 % when triplicate amplification is performed and an FPR of 20 % for single amplification [1]. We thus compared tropism prediction results obtained via single and triple amplification using the FPRs reported by the guidelines. This analysis resulted in nine cases of discordant tropism prediction, and in particular in eight reclassification from CXCR4 to CCR5 tropism. This result suggests that the use of an FPR of 20 % as a cutoff for tropism determination can substantially reduce the number of patients assessed to be candidates for treatment with the CCR5 antagonist.

We found that a FPR of <5.75 was associated not only with a lower CD4 cell count at the time of V3 sequencing but also with a lower increase in the CD4 cell count during HAART despite virological success being achieved. These results are in line with a previous study in viremic patients showing that a FPR of ≤2 % defines a viral population associated with a low CD4 rank, potentially greater cytopathic effect, and more advanced disease [16]. Interestingly, we also found an inverse correlation between FPR and CD8 + cell count. In particular, we found that a FPR of <5.75 was associated with an higher CD8 cell count at the time of V3 sequencing. This could be explained by the proven interaction between CD8 + T cell antiviral activity and the rate of CD4 + T-cell decline [17], and also by the ability of CD8 + T-lymphocytes to suppress CXCR4 HIV-1 replication [18]. Thus, a finer use of genotypic tropism testing might be useful to gain information regarding the pathogenic potential of HIV.

In conclusion, we have developed a population-based sequencing assay for genotypic HIV-1 tropism determination on HIV-DNA. Using this assay, it is possible to examine both PBMC and whole blood samples from patients with undetectable viral loads. The assay also covers the spectrum of HIV-1 group M subtypes. The assay is robust and can be set up easily in any standard clinical laboratory. Our results support the feasibility and consistency of genotypic tropism testing on HIV-DNA as a laboratory tool, potentially assisting the selection of patients with suppressed plasma HIV-1 RNA as candidates for CCR5-antagonist treatment as part of switching, simplification, or intensification strategies.

## Appendix 1

The complete list of centers and members participating in the DIVA programme is as follows: “San Raffaele” Hospital (Milan): Adriano Lazzarin, Massimo Clementi, Silvia Nozza, Filippo Canducci, Enzo Boeri, Angela Rosa Pignataro, Francesco Santoro; “L. Sacco” Hospital (Milan): Giuliano Rizzardini, Massimo Galli, Valeria Micheli; “S. Paolo” Hospital *(Milan)*: Antonella D’Arminio Monforte; Busto Arsizio Hospital (Busto Arsizio [MI]): Tiziana Quirino; “S. Gerardo” Hospital (Monza [MI]): Andrea Gori, Laura Vecchi; Ospedali Riuniti (Bergamo): Franco Maggiolo, Anna Paola Callegaro; IRCCS Policlinico S. Matteo (Pavia): Renato Maserati, Fausto Baldanti, Stefania Paolucci, Roberto Gulminetti; Ospedali Civili di Brescia: Francesco Castelli, Franco Gargiulo; University of Turin (Turin): Giovanni Di Perri, Valeria Ghisetti, Tiziano Allice; University of Padova: Saverio Parisi, Renzo Scaggiante; Policlinico “S. Orsola-Malpighi” (Bologna): Marco Borderi, Maria Carla Re, Isabella Bon; “San Martino” Hosptial (Genova): Claudio Viscoli, Antonio Di Biagio, Bianca Bruzzone, Renata Barresi, Silvia Calderisi; Policlinico of Modena (Modena): Cristina Mussini, William Gennari, Monica Pecorari; Marche Politechnic University Medical School (Ancona): Andrea Giacometti, Alessia Monachetti, Patrizia Bagnarelli; “S.M. Annunziata” Hospital (Firenze): Francesco Mazzotta, Massimo Di Pietro; “Careggi” Hospital (Firenze): Francesco Leoncini, Gaetana Sterrantino, Canio Martinelli; AOUPisana: Maria Linda Vatteroni; University of Siena (Siena): Maurizio Zazzi, Andrea De Luca, Genni Meini; University of “Tor Vergata” (Rome): Carlo Federico Perno, Valentina Svicher, Claudia Alteri, Fabioloa Di Santo, Michela Pollicita, Massimo Andreoni Gaetano Maffongelli, Marco Montano, Alessandra Nori; I·N.M.I. “L. Spallanzani” (Rome): Andrea Antinori, Carlo Federico Perno, Roberta D’Arrigo, Maria Rosaria Capobianchi; University of Rome “La Sapienza” (Rome): Vincenzo Vullo, Guido Antonelli, Ombretta Turriziani; Catholic University “Sacro Cuore” (Rome): Roberto Cauda, Emanuela Colafigli, Giovanni Fadda, Rosaria Santangelo; Pescara General Hospital: Giustino Parruti; Ospedale Cotugno: Antonio Chirianni, Giuseppe Di Nicuolo; University of Foggia and Bari: Gioacchino Angarano, Laura Monno, Annalisa Saracino, Grazia Punzi.

## Appendix 2

The cellular line 8E5, containing one copy of HIV-1 DNA integrated for each cell, was used to build a standard curve of seven dilutions (75,000–37,500–3,750–375–37.5–3.75–1.87 copies). The sensitivity of PCR is one copy of HIV-1 DNA per reaction (one copy per reaction = 3.3 copies/10^6^ PBMCs). The HIV-1 DNA target was hybridized with the TaqMan probe and read on channel F1/F2 of the LightCycler system (Roche Molecular Systems, Indianapolis, IN). To verify DNA integrity, the reagents of the LC Control DNA kit (Roche) were used, amplifying a 110-bp fragment of human b-globin in the same reaction. This second internal control target was hybridized with the FRET probe and read on channel F3/F2 of the LightCycler system. To verify the accuracy of the Real Time PCR results, different HIV-1 DNA standards (AIDS Research and Reference Reagent Program, DAIDS, NIAID, NIH: PCR Panel 001 from Dr. Shirley Kwok and Dr. Cindy Christopherson, Roche Molecular Systems) were also quantified. The quantification of total HIV-1 DNA was centralized and performed at the laboratories of the University of Tor Vergata. Each center participating to the DIVA project kept the samples at −70 °C (or at −20 °C if not available) for up to 4 weeks. Samples were then sent to the University of Tor Vergata in dry ice.
